# Immune-mediated hepatitis induced by immune checkpoint inhibitors: Current updates and future perspectives

**DOI:** 10.3389/fphar.2022.1077468

**Published:** 2023-01-09

**Authors:** Zherui Liu, Yun Zhu, Huan Xie, Zhengsheng Zou

**Affiliations:** ^1^ Senior Department of Hepatology, the Fifth Medical Center of PLA General Hospital, Beijing, China; ^2^ Peking University 302 Clinical Medical School, Beijing, China; ^3^ Medical School of Chinese PLA, Beijing, China

**Keywords:** cancer, immunotherapy, immune checkpoint inhibitors, immune-related adverse events, drug-induced liver injury, hepatitis

## Abstract

In recent years, cancer immunotherapy has made remarkable achievements. Immune checkpoint inhibitors (ICIs) have been used successfully in several types of cancer in the past decade. However, expanded indication and increased use of Immune checkpoint inhibitors have resulted in increased reports of toxicity called immune-related adverse events (irAEs). Due to the unique immunological characteristics of the liver, a hepatic immune-related adverse events has also been reported, which is usually termed Immune-mediated hepatitis (IMH). So far, it is generally considered that the mechanism of IMH induced by Immune checkpoint inhibitors is mainly the overactivation of T cells. It has been reported that the incidence of IMH ranges from 1% to 15%. Because of the lack of specific markers, a diagnosis of exclusion of IMH is critical. Although most IMH is mild and recoverable, several death cases have been reported, which has been increasingly concerned. This review summarizes the current understanding of the pathophysiology, epidemiology, diagnosis, management and prognosis of IMH caused by Immune checkpoint inhibitors. It also discusses the controversial issues in IMH, such as the role of liver biopsy, grading criteria, risk factors, rational treatment strategies with steroids, and the timing of Immune checkpoint inhibitors rechallenging, which may provide helpful information for IMH in future clinical practice.

## Introduction

In the past decade, immune checkpoint inhibitors (ICIs) have developed rapidly in the application of advanced malignancies (Bagchi et al., 2021). According to the targets of immune checkpoint molecules which act as negative regulators of T cells function in cancer immunological process, there are three main types of ICIs so far: cytotoxic T-lymphocyte-associated protein 4 (CTLA-4), programmed cell death protein 1 (PD-1) and programmed death-ligand 1 (PD-L1) (Qin et al., 2019; Kotanides et al., 2020). ICIs, the monoclonal antibodies of these molecules, have been exploited to block these immune checkpoint molecules, enhance T cells function and finally recover anti-tumor activity in the host. Since a CTLA-4 inhibitor, ipilimumab, has been approved by America food and drug administration against advanced-stage melanoma in 2011 (Hodi et al., 2010), ICIs have become a hotspot and have revoluted treatments of various cancers (Table 1).

**TABLE 1:** Immune checkpoint inhibitors and their indications.

Target	Drug name	Indications	Time to market
CTLA-4	Ipilimumab (Yervoy)^a^ ^,^ ^b^	Melanoma, advanced RCC, MSI-H or dMMR CRC, HCC, metastatic NSCLC, MPM, esophageal cancer	2011
PD-1	Nivolumab (Opdivo)^a^ ^,^ ^b^	Melanoma, NSCLC, MPM, advanced RCC, classical hodgkin lymphoma, HNSCC, urothelial carcinoma, MSI-H or dMMR CRC, HCC, esophageal cancer, gastric cancer, gastroesophageal junction cancer, and esophageal adenocarcinoma	2014
PD-1	Pembrolizumab (Keytruda)^a^ ^,^ ^b^	Melanoma, NSCLC, HNSCC, classical hodgkin lymphoma, PMBCL, urothelial carcinoma, MSI-H or dMMR CRC, gastric cancer, esophageal cancer, cervical cancer, HCC, MCC, RCC, endometrial carcinoma, TMB-H solid tumors, cutaneous squamous cell carcinoma, TNBC	2014
PD-L1	Atezolizumab (Tecentriq)^a^ ^,^ ^b^	Locally advanced or metastatic urothelial carcinoma, metastatic NSCLC, SCLC, HCC, melanoma	2016
PD-L1	Avelumab (Bavencio)^a^	Metastatic MCC, locally advanced or metastatic urothelial carcinoma, advanced RCC	2017
PD-L1	Durvalumab (Imfinzi)^a^ ^,^ ^b^	NSCLC, SCLC	2017
PD-1	Toripalimab^b^	Melanoma, metastatic nasopharyngeal carcinoma, metastatic urothelial carcinoma	2018
PD-1	Sintilimab^b^	Classical hodgkin lymphoma, NSCLC, HCC	2018
PD-1	Cemiplimab (Libtayo)^a^	Cutaneous squamous cell carcinoma, basal cell carcinoma, NSCLC	2018
PD-1	Camrelizumab^b^	Classical hodgkin lymphoma, advanced HCC, advanced or metastatic esophageal squamous carcinoma, nasopharyngeal carcinoma	2019
PD-1	Tislelizumab^b^	Classical hodgkin lymphoma, metastatic urothelial carcinoma, metastatic NSCLC, HCC, esophageal squamous carcinoma	2019
PD-1	Penpulimab^b^	Classical hodgkin lymphoma	2021
PD-1	Zimberelimab^b^	Classical hodgkin lymphoma	2021
PD-L1	Envafolimab^b^	MSI-H or dMMR CRC	2021
PD-L1	Sugemalimab (Cejemly)^b^	NSCLC	2021

^**a**^Approved by U.S. Food and Drug Adminstration.

^**b**^Approved by National Medical Products Administration (China).

CTLA-4, cytotoxic T-lymphocyte associated protein four; PD-1, programmed cell death protein 1; PD-L1, programmed death-ligand 1; RCC, renal cell carcinoma; MSI-H, microsatellite instability-high; dMMR, deficient mismatch repair; CRC, colorectal cancer; HCC, hepatocellular carcinoma; NSCLC, non-small cell lung cancer; MPM, malignant pleural mesothelioma; HNSCC, head and neck squamous cell cancer; SCLC, small Cell Lung Cancer; PMBCL, primary mediastinal large B-cell lymphoma; MCC, merkel cell carcinoma; TMB-H, tumor mutational burden-high; TNBC, triple-negative breast cancer.

However, with the wide application of ICIs, several unexpected immunological and inflammatory events, termed immune-related adverse events (irAEs), have been reported (Michot et al., 2016). It has been demonstrated that irAEs result from overactive immune response, which can affect almost any organ, especially skin, liver, endocrine and gastrointestinal tract (Regev et al., 2020). As an essential organ of drug metabolism, liver is one of the frequently affected organs in cancer immunotherapy and its injury caused by ICIs is usually termed immune-mediated hepatitis (IMH). It has been reported that IMH is the third most frequent adverse event (5%–10%), after dermatologic toxicity (44%–68%) and gastrointestinal adverse reactions (35%–50%) (Kroner et al., 2019). In recent years, the incidence of IMH has increased. Although most IMH cases are mild, there is a risk of acute liver failure and even death if the diagnosis or management is not properly (Vozy et al., 2019; Yamamoto et al., 2021), especially in hepatocellular carcinoma (HCC) patients on a background of chronic liver diseases. Furthermore, inappropriate interventions of IMH may cause the failure of cancer immunotherapy. Therefore, IMH has become an increasing concern and a large amount of clinical data has accumulated.

This review aims to discuss the pathophysiology, epidemiology, diagnosis, management and prognosis of IMH caused by ICIs and provide references for the clinical application of ICIs.

## Underlying mechanisms of IMH

**FIGURE 1:**
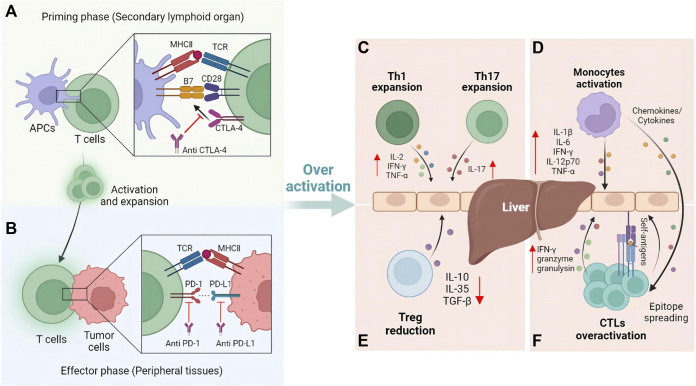
Mechanisms of T cells activation and immune-mediated hepatitis caused by ICIs. **(A)** Blockade of CTLA-4 activates T cells at the priming phase. **(B)** Further anti-tumor effect induced by the blockade of PD-1 and PD-L1 occurs in the effector phase. Once liver self-tolerance impairs, immune cells such as **(C)** Th cells, **(D)** Monocytes, **(E)** Treg cells, and **(F)** cytotoxic T cells will be involved in the pathophysiological process of immune-mediated hepatitis.

The critical step for ICIs in cancer immunotherapy is the activation of T cells. As mentioned above, CTLA-4, PD-1 and PD-L1 are three current ICIs targets. However, the mechanisms of these ICIs are different. It has been demonstrated that the CTLA-4 inhibitors play a role in the initial phase, while PD-1 and PD-L1 inhibitors are involved in the effector phase (Buchbinder and Desai, 2016). In the initial stage, CTLA-4 on T cells competitively binds with CD28 to B7-1 and B7-2 on antigen presenting cells (APCs), inhibiting the activation of T cells (Figure 1A). CTLA-4 inhibitors can enhance T cell activation by binding to CTLA-4 and increasing CD28 and B7 costimulatory signals (Yang et al., 2020). In the effector phase, binding of PD-1 on T cells and PD-L1 on tumor cells inhibits T cells activation and allows tumor cells to evade immune surveillance (Figure 1B). Similar to CTLA-4 inhibitors, PD-1/PD-L1 inhibitors can block this binding and enhance the anti-tumor effect of T cells (Peeraphatdit et al., 2020).

To date, the mechanism of IMH caused by ICIs has not been fully elucidated. However, the unique immunological features of the liver are crucial to the pathogenesis of IMH. Portal circulation connects the liver to the intestines, thus making the liver the first site to detoxify the blood entering the portal circulation and to process many antigen exposures. Therefore, the liver has evolved specific immune mechanisms to protect the organism from pathogens while maintaining a state of immunotolerance to harmless antigens from the intestine (Crispe, 2014). As one of the key mechanisms of liver immunotolerance, PD-L1 expressed on hepatic non-parenchymal cells including hepatic stellate cells, Kupffer cells and dendritic cells, together with CTLA-4 expressed on CD4^+^ Treg cells, protect the liver from autoimmune responses to antigens by downregulating effector T cells (Makarova-Rusher et al., 2015). However, due to the use of ICIs blocking these key modulatory pathways, T cells may be overactive and the immune tolerance of the liver can be broken, making it susceptible to acute inflammatory response, which further induces hepatitis (Gudd and Possamai, 2022).

Current evidence suggests several primary mechanisms of IMH: Firstly, expansion of T helper cells in ICIs therapy such as Th1 and Th17 cells increase the levels of proinflammatory cytokines (IL-2, IFN-γ, TNF) production, which can go on to activate cytotoxic T lymphocytes (Figure 1C), as well as innate immune cells such as macrophages and natural killer cells (Gudd et al., 2021). Secondly, ICIs induce the activation of monocytes and lead to formation of an inflammatory environment related to IMH (Figure 1D) (Gudd et al., 2021). Thirdly, reduction of regulatory T cells (Treg) caused by ICIs can reduce anti-inflammatory cytokines such as interleukin (IL) -10, IL-35, TGF-β and modulate the interaction between adaptive-innate immunity (Figure 1E) (Vignali et al., 2008). Additionally, clonal expansion of CD8^+^ T cells and epitope spreading is another mechanism of IMH (Vanderlugt and Miller, 2002; Das et al., 2015; Riaz et al., 2017). ICIs could stimulate the proliferation of CD8^+^ T cells to overcome immune tolerance, which could further upregulate proliferative and cytotoxic genes such as IFN-γ, granzyme and granulysin. At the same time, epitope spreading causes an indiscriminate immune reaction to self-antigens (Figure 1F).

In addition, due to the high exogenous antigens exposure such as LPS in the liver, Kuffer cells and liver sinusoidal endothelial cells (LSECs) express the adhesion molecules intercellular adhesion molecule 1 (ICAM-1) and vascular cell adhesion molecule 1 (VCAM-1). These continuously expressed adhesion molecules and the slow blood flow in the hepatic sinusoids promote the interaction of activated CD8^+^ T cells in the systemic circulation with Kuffer and LSECs, leading to the retention of activated CD8^+^ T cells in the liver (Mehal et al., 1999; John and Crispe, 2004). Upon retention, these cells bind and secrete IFN-γ through their FasL molecules and Fas expressed by Kuffer cells, inducing TNF-α secretion by Kuffer cells (Murray and Crispe, 2004), which would induce hepatocytes sensitive and susceptible to Fas-induced and IFN-γ-mediated apoptosis (Horras et al., 2011; Faletti et al., 2018), leading to hepatocyte injury. Although this hypothetical mechanism may not answer why IMH occurs in only a subset of patients on ICI treatment and not in most patients, this hypothesis provides a possible mechanism of IMH, further studies are still needed.

## Incidence

The incidence of IMH is mainly counted through the reports of irAEs in clinical trials. Up to date, most trials defined the occurrence and grades of irAEs based on the common criteria for adverse events (CTCAE), which was used by referring to the elevations of aminotransferase (ALT), aspartate transaminase (AST), alkaline phosphatase (ALP) and total bilirubin based on the upper limit of normal (ULN).

The reported incidence of IMH varies according to the different agents, dosages and indications (Regev et al., 2020). It has been described that the incidence of all grades of IMH widely ranges from 1% to 15%, and the incidence of grade 3 or four ranges from 1% to 10% (Table 2). The incidence of IMH caused by CTLA-4 inhibitors (2%–15%) usually demonstrates an increased risk compared to those using PD-1 (0%–3%) or PD-L1 (0%–6%) inhibitors (Weber et al., 2009; Robert et al., 2015; Choueiri et al., 2018; Aamdal et al., 2022). Meanwhile, a higher dose of CTLA-4 inhibitors appears to increase the incidence of ICIs induced IMH. For melanoma patients who received ipilimumab, monotherapy with high doses (10 mg/kg) may cause an increased incidence compared to lower doses (3 mg/kg) (Wolchok et al., 2010; Robert et al., 2015). Furthermore, combination therapy seems more likely to cause IMH than monotherapy. In a phase Ⅲ clinical trial of CheckMate 067, patients with advanced melanoma who received ipilimumab plus nivolumab were reported a higher incidence of IMH than those who received ipilimumab or nivolumab alone (Wolchok et al., 2022). In another clinical trial, KEYNOTE-598, patients with non-small-cell lung cancer who received pembrolizumab plus ipilimumab reported a approximate incidence of all grades of IMH compared to those who received pembrolizumab alone. However, the incidence of grade 3/4 IMH was higher in the combined treatment group than in the monotherapy group, suggesting that combined therapy of ICIs may be associated with more severe IMH (Boyer et al., 2021). Furthermore, compared to IMH caused by ICIs in other tumors, the incidence of IMH in patients with HCC may be slightly higher (Zhu et al., 2018; Sangro et al., 2020). Similar to other tumors, the incidence of IMH with combined therapy is much higher than those received monotherapy in HCC (Zhu et al., 2018; Finn et al., 2020a; Yau et al., 2020). The background of chronic liver disease and the influence of the primary location of HCC may partly explain the higher incidence of IMH in HCC. However, the incidence of IMH may be overestimated due to causes other than ICIs, such as other drugs, viral reactivation or tumor progression. More rigorous assessment and differential diagnosis need to be developed.

**TABLE 2:** Incidence of immune-mediated hepatitis according to different treatment regimens with immune checkpoint inhibitors.

Pathway	Agent	Dose	Indication	Patients, n	Incidence of all grades of IMH, n (%)	Incidence of grade 3/4 of IMH, n (%)	Ref
CTLA-4	Ipilimumab	3 mg/kg every 3 weeks for four dosages	Melanoma	151	4 (2.65)	2 (1.32)	Aamdal et al. (2022)
		3 mg/kg every 3 weeks for four dosages	Melanoma	256	3 (1.17)	1 (0.39)	Robert et al. (2015)
		10 mg/kg every 3 weeks for four dosages	Melanoma	57	8 (14.04)	7 (12.28)	Weber et al. (2009)
		10 mg/kg every 3 weeks for four dosages	Melanoma	71	2 (2.82)	2 (2.82)	Wolchok et al. (2010)
PD-1 PD-L1	Nivolumab	3 mg/kg every 2 weeks	Melanoma	313	1 (0.32)	1 (0.32)	Wolchok et al. (2022)
		240 mg every 2 weeks	Advanced NSCLC	391	5 (1.28)	4 (1.02)	Paz-Ares et al. (2022)
		480 mg every 4 weeks	Melanoma	359	9 (2.51)	4 (1.11)	Tawbi et al. (2022)
	Cemiplimab	350 mg every 3 weeks	Advanced NSCLC with PD-L1 of ≥50%	355	2 (0.56)	2 (0.56)	Sezer et al. (2021)
		350 mg every 3 weeks	Recurrent or metastatic cervical carcinoma	300	0 (0.00)	4 (1.33)	Tewari et al. (2022)
		3 mg/kg every 2 weeks	cSCC with or without metastatic	78	1 (1.28)	1 (1.28)	Migden et al. (2020)
	Pembrolizumab	200 mg every 3 weeks	Melanoma	509	9 (1.77)	7 (1.38)	Eggermont et al. (2018)
		10 mg/kg every 3 weeks for four dosages	Melanoma	277	5 (1.81)	5 (1.81)	Robert et al. (2015)
		200 mg every 3 weeks	HCC	104	3 (2.88)	3 (2.88)	Zhu et al. (2018)
		200 mg every 3 weeks	HCC	279	5 (1.79)	4 (1.43)	Finn et al. (2020a)
	Atezolizumab	1200 mg every 3 weeks	Advanced NSCLC	68	1 (1.47)	1 (1.47)	Cho et al. (2022)
		1200 mg every 3 weeks	Muscle-invasive urothelial carcinoma	390	36 (9.23)	9 (2.31)	Bellmunt et al. (2021)
	Avelumab	10 mg/kg every 2 weeks	Clear-cell renal-cell carcinoma	55	3 (5.45)	2 (3.64)	Choueiri et al. (2018)
	Durvalumab	1500 mg every 4 weeks	Urothelial carcinoma	345	1 (0.29)	1 (0.29)	Powles et al. (2020)
Combination Therapy	Nivolumab plus ipilimumab	1 mg/kg nivolumab plus 3 mg/kg ipilimumab every 3 weeks for four dosages	Melanoma	313	7 (2.23)	5 (1.60)	Hodi et al. (2018)
		1 mg/kg nivolumab plus 3 mg/kg ipilimumab once every 3 weeks for four dosages, followed by nivolumab 3 mg/kg once every 2 weeks	Melanoma	313	1 (0.32)	1 (0.32)	Wolchok et al. (2022)
		nivolumab 1 mg/kg plus ipilimumab 3 mg/kg every 3 weeks for four dosages, followed by nivolumab 240 mg every 2 weeks	HCC	49	10 (20.41)	10 (20.41)	Yau et al. (2020)
		Nivolumab3 mg/kg plus ipilimumab 1 mg/kg every 3 weeks for four dosages, followed by nivolumab 240 mg every 2 weeks	HCC	49	6 (12.24)	5 (10.20)	Yau et al. (2020)
		Nivolumab 3 mg/kg every 2 weeks plus ipilimumab 1 mg/kg every 6 weeks	HCC	49	3 (6.12)	3 (6.12)	Yau et al. (2020)
	Pembrolizumab plus ipilimumab	200 mg pembrolizumab every 6 weeks every 3 weeks, followed by ipilimumab 1 mg/kg	Metastatic NSCLC	282	5 (1.77)	4 (1.42)	Boyer et al. (2021)
		2 mg/kg pembrolizumab every 3 weeks, followed by 1 mg/kg ipilimumab every 3 weeks for four dosages, followed by 2 mg/kg pembrolizumab every 3 weeks	Melanoma	153	15 (9.80)	9 (5.88)	Long et al. (2017)

CTLA-4, cytotoxic T-lymphocyte associated protein four; PD-1, programmed cell death protein 1; PD-L1, programmed death-ligand 1; NSCLC, non-small-cell lung cancer; HCC, hepatocellular carcinoma; cSCC, cutaneous squamous cell carcinoma.

In addition, it is worth noting that there are some commonalities between other irAEs and IMH. The incidence of other irAEs was also associated with different treatment strategies. It has been reported that the incidence of rash, colitis and diarrhea is higher in patients treated with anti-CTLA-4 than in patients treated with PD-1 (33% vs. 26%, 12% vs. 1%, 33% vs. 20%) (Kroner et al., 2019). The risk of non-hepatic irAEs has been demonstrated to be dose dependent in anti-CTLA-4 agents (Ascierto et al., 2017). Meanwhile, compared to monotherapy, combined immunotherapy has higher incidence in most of irAEs and more than 60% of patients treated with combined therapy have been reported to occur severe irAEs (Wolchok et al., 2017; Esfahani et al., 2019). Furthermore, nearly half of IMH patients are reported to have concomitant non-hepatic irAEs such as pneumonia, pituitary inflammation, hyperthyroidism and pancreatitis, which may precede the diagnosis of IMH (De Martin et al., 2018; Huffman et al., 2018).

Although IMH occurs less commonly than some other non-hepatic irAEs, fatal cases have been observed in both clinical trials and post marketing phase. A meta-analysis investigated the fatality rates caused by ICIs, which indicated that in 613 reported fatal cases, 124 were secondary to IMH. Furthermore, in this study, of all fatal cases, 31 (5.1%) in the ipilimumab group, 74 (12.1%) in the anti-PD-1/PD-L1 group and 19 (3.1%) in the combined therapy group were caused by IMH. The study further analyzed the patients with melanoma from seven international academic medical centers and found that 21 fatal cases were reported, of which 5 (23.8%) cases were caused by IMH, followed by myocarditis (28.6%) and colitis/enteritis (28.6%) (Wang et al., 2018). These studies suggest that IMH accounts for a high proportion of fatal irAEs, which is noteworthy and has important clinical significance.

## Risk factors

Although certain risk factors have been associated with irAEs during ICIs therapy, the risk factors associated with IMH have not been fully elucidated (Yang et al., 2020). There are several risk factors have been demonstrated until now, such as the therapeutic strategy of ICIs, background in chronic liver diseases, and some other factors demonstrated by several clinical reports.

### Therapeutic strategy

From the perspective of the treatment strategy of ICIs, it has been reported that the incidence of irAEs in monotherapy of anti-CTLA-4 is higher than that in anti-PD-1 or PD-L1, which suggests that the types of ICIs may be a risk factor in IMH. Furthermore, the risk of incidence of IMH is correlated with the dosage of ICIs. In a study of ipilimumab for melanoma, serious hepatic adverse events were more common at 10 mg/kg compared to the dosage of 3 mg/kg (30% vs. 0%) (Wolchok et al., 2010). Additionally, ipilimumab plus nivolumab combination therapy and previous ICI treatment are two independent risk factors for IMH, respectively (Kitagataya et al., 2020; Yamamoto et al., 2021).

As for the drugs other than ICIs, it has been reported that acetaminophen was associated with a 2.1-fold increased risk of all grades of IMH and the use of 3-hydroxy-3-methyl-glutaryl-coenzyme reductase inhibitors was associated with a 4.7-fold increased risk of grade 3 or higher IMH compared with untreated (Cho et al., 2021).

### Chronic liver diseases

For patients with a background in chronic liver diseases, the incidence of IMH is higher than that of patients without liver dysfunction (Sangro et al., 2020). However, a clinical trial has reported no relation between the occurrence of IMH and the background of viral hepatitis in HCC patients who received nivolumab monotherapy (El-Khoueiry et al., 2017). A retrospective study on a total of 135 patients who received PD-1 inhibitors has reported 8 cases occurred IMH, two cases of combined non-alcoholic fatty liver disease (NAFLD) and one case of combined alcoholic liver disease, which suggests that some liver disease other than chronic viral hepatitis may also increase the risk of IMH (Sawada et al., 2020). Further analysis in the study has shown a significant correlation between NAFLD and IMH (hazard ratio [HR] = 29.34, *p* = 0.003). Furthermore, several studies have demonstrated that patients with autoimmune disorders such as thyroiditis or rheumatological have a higher risk of IMH during ICIs therapy (Johnson et al., 2016; Abdel-Wahab et al., 2018). However, there is still no available data supporting this tendency in autoimmune hepatitis, which needs further investigation.

### Other factors

For sex, a retrospective study confirmed that male (HR = 1.608, *p* < 0.05) was an independent risk factor for IMH (Cho et al., 2021). However, another study has reported that females are significantly associated with higher grade IMH compared to males, which still exists a divergence and further studies are necessary to draw a definite conclusion (Kitagataya et al., 2020). Furthermore, for age, a study by Cho et al. demonstrated that patients younger than 65 years old (HR = 1.527, *p* < 0.05) was another independent risk factor for IMH (Cho et al., 2021), which may be due to the immunosenescence as people age (Nishijima et al., 2016).

Secondly, as for the types of cancer, a Japanese study reported that malignant melanoma was significantly and independently associated with increased risk of IMH (odd ratio [OR] = 11.6, *p* = 0.002) (Yamamoto et al., 2021), which suggested that comprehensive and systematic evaluation should be carried out in malignant melanoma patients who received ICIs therapy to reduce the risk of IMH. Additionally, the risk of IMH has been reported to be associated with patients with primary liver cancer. It has shown higher elevations of ALT, AST, total bilirubin, and more severe grade of IMH in patients with primary liver cancer compared to patients with other solid tumors (Fu et al., 2021), which suggests that more concern should be paid to the occurrence of IMH in HCC patients during ICIs administration.

Furthermore, fever over 38°C within 24 h of initial ICI treatment was also identified as another risk factor for IMH (HR = 6.21, *p* < 0.001) (Mizuno et al., 2020). In sum of these studies, risk factors of IMH need to be further investigated, which may be helpful to reveal the underlying mechanisms of IMH caused by ICIs and to improve the diagnosis and management of IMH in clinical practice in the future.

## Diagnosis

Although most cases of IMH are asymptomatic, a few patients may present with fatigue, abdominal discomfort, fever, rash, and rarely jaundice (Huffman et al., 2018; Riveiro-Barciela et al., 2020). Acute liver failure is also rarely present in the initial stage of IMH. Furthermore, the clinical presentation is demonstrated to vary in different types of ICIs. It has been reported that fever is more prevalent in CTLA-4 inhibitors than in PD-1 and PD-L1 inhibitors (De Martin et al., 2018). The pattern of IMH commonly presents the type of hepatocellular injury, while a cholestatic or mixed liver injury pattern may also be observed, which is more commonly secondary to PD-1 and PD-L1 inhibitors than CTLA-4 inhibitors (De Martin et al., 2018; Imoto et al., 2019).

Abnormal elevations of serum liver enzymes in liver function tests are usually indexed in the diagnosis of IMH. Elevations of ALT or AST more than two times ULN should be concerned. Sometimes it should also be concerned mild to moderate elevation of serum ALP >2.5 × ULN, and abnormal elevation of total bilirubin >1.5 × ULN. Since the IMH is usually asymptomatic or has non-specific symptoms, many cases are diagnosed during monitoring during ICI therapy. Furthermore, specific biomarkers of IMH have not been elucidated. Although recent studies have demonstrated that human leukocyte antigen and IL-6 are susceptible to liver injury induced by ICIs, there is no specificity in IMH, which needs more studies to verify (Chowell et al., 2018; Valpione et al., 2018). For time to onset of IMH, it has been reported that the onset time of IMH is between 4 and 12 weeks or after 3 times of ICIs infusion, the onset time of IMH induced by CTLA-4 inhibitors is sooner than that induced by PD-1 and PD-L1 inhibitors (De Martin et al., 2018).

The 2019 European Association for the Study of the Liver (EASL) guideline for drug-induced liver injury (DILI) classified IMH as a special type of DILI (European Association for the Study of the Liver, 2019). Similar to idiosyncratic DILI, IMH is a diagnosis of exclusion and it is essential to assess the causality in patients with abnormal liver function tests to confirm IMH (Regev et al., 2014). The Roussel Ucalf Causality Assessment Method (RUCAM) scale is a well-established tool to assess the likelihood of DILI and is recommended to assist the diagnosis of IMH by some hepatologic experts (Danan and Teschke, 2015), which includes the assessment of onset time after therapy, the course of liver enzymes after drugs cessation, response to drug re-exposures, alcohol use, age, and concomitant drugs (Hoofnagle and Bjornsson, 2019). However, the RUCAM scale in IMH diagnosis is less application in the diagnosis of IMH. It should be further verified in clinical practice to evaluate whether RUCAM is suitable for the IMH diagnosis.

### Differential diagnosis

The differential diagnosis of IMH is still challenging as the existing a lot etiologies of abnormally elevated liver enzymes, which mainly include drugs other than ICIs, viral infection, autoimmune and metabolic diseases, tumor-related causes, biliary diseases, musculoskeletal and cardiovascular system diseases (Shantakumar et al., 2016; Ricart, 2017). Therefore, it is important to comprehensively assess other common differentials to avoid the inappropriate interruption of effective anticancer therapy or unnecessary interventions in patients suspected IMH during ICIs therapy.

Identification of the above differential causes of liver injury during ICIs therapy requires a detailed medication history. It is noteworthy that chemotherapeutic drugs combined with ICIs, such as dacarbazine, carboplatin, and bevacizumab, which may also cause liver injury during cancer immunotherapy (Reck et al., 2013; Fashoyin-Aje et al., 2019; Finn et al., 2020b). Furthermore, dietary supplements, herbal, as well as alcohol can also induce a non-immune mediated hepatitis. Another cause of liver injury that deserves mention is liver metastasis as ICIs are usually for patients with advanced malignancies. A cohort of 491 patients who received pembrolizumab reported 14.3% incidence of liver injury, however, more than half patients were found with liver metastasis, which suggests that liver metastasis may be the cause of the liver injury rather than ICIs (Tsung et al., 2019). Other chronic liver diseases such as hepatic viral infection should also be concerned. Additionally, some extra-hepatic causes of elevation of liver enzymes also need to be considered, such as myocarditis, myositis, and bone or other organ metastasis (Regev et al., 2014; Touat et al., 2018). A detailed differential diagnosis and related tests in IMH diagnosis are listed in Table 3.

**TABLE 3:** Differential diagnosis and recommended tests in immune-mediated hepatitis diagnosis.

Etiology	Differential diagnosis	Related tests
Drugs other than ICIs	Concomitant anti-tumor medications; Complementary and herbal medications; acetaminophen toxicity	Medication history
Viral infection	a) Hepatic virus infection (HAV, HBV, HCV, HEV); b) Reactivation of HBV; c) CMV infection; d) EBV infection; e) HSV infection	a) anti-HAV IgM, HBsAg, anti-HBc IgG, anti-HBc IgM, HBV DNA, anti-HCV, HCV RNA, anti-HEV IgG, anti-HEV IgM, HEV RNA; b) HBV DNA; c) anti-CMV IgM, CMV DNA; d) anti-EBV IgM, EBV DNA; e) anti-HSV IgM, HSV DNA
Alcohol related	Alcoholic hepatitis	Alcohol intake history
Autoimmune disease	Autoimmune hepatitis	ANA, ASMA, anti-LKM-1, anti-LC-1, anti-SLA/LP, pANCA, serum IgG, IgM, IgA
Metabolic disease	Non-alcoholic fatty liver disease	Metabolic risk factor, imaging of hepatic steatosis
Tumor related	Hepatic metastasis or HCC progression	Hepatic imaging (ultrasonography, CT scan, MRI)
Biliary disease	Biliary obstruction; Gallstones; Cholecystitis; Cholangitis	Hepatobiliary imaging (ultrasonography, CT scan, MRI, MRCP)
Genetic disease	Wilson’s disease	Blood ceruloplasmin, serum copper, slit lamp eye examination for Kayser-Fleischer rings, genetic testing
Systemic infection	Sepsis	Blood pressure, complete blood count, procalcitonin, blood or urine cultures
Musculoskeletal system	Muscle injury (mostly myositis); Rhabdomyolysis	Serum CK; CK-MB
Cardiovascular system	Myocarditis; Portal-vein/hepatic vein thrombosis; Ischemic or congestive hepatic injury	Imaging and clinical history (Blood pressure, pulse, electrocardiogram, echocardiogram)

ICIs, immune checkpoint inhibitors; HAV, hepatitis A virus; HBV, hepatitis B virus; HCV, hepatitis C virus; HEV, hepatitis E virus; CMV, cytomegalovirus; EBV, epstein barr virus; HSV, herpes simplex virus; ANA, anti-nuclear antibody; ASMA, anti-smooth muscle antibody; CK, creatine kinase; CK-MB, creatine kinase-MB; CT, computed tomography; MRI, magnetic resonance imaging; MRCP, magnetic resonance cholangiopancreatography; LKM-1, liver kidney microsomal type 1; LC-1, liver cytosol type 1; SLA, soluble liver antigen; LP, liver pancreas; pANCA, perinuclear anti-neutrophil cytoplasmic antibodies; HCC, hepatocellular carcinoma.

### Pathologic diagnosis

Liver biopsy is commonly unnecessary for diagnosis as the feature that IMH is a diagnosis of exclusion and is often reflected on liver tests. At present, it is recommended that a liver biopsy may reserve for patients with more severe than grade 2 (Sangro et al., 2020). As liver biopsy is unnecessary in most patients, there are few histological appearance data during IMH caused by ICIs. Common histopathology findings from reported cases are mainly mononuclear inflammation, including periportal inflammation with or without interface hepatitis, diffuse panlobular inflammation with prominent perivenular infiltrate, confluent necrosis, and rarely cholestatic injury which appears a mononuclear infiltrate in portal tracts that are centered around bile ducts and bile ductular proliferation (Kleiner and Berman, 2012; Kim et al., 2013; Kawakami et al., 2017; Zhang et al., 2020). Immune cells in liver tissues of patients with IMH consist of predominantly CD8^+^ T cells and eosinophils, less frequently CD4^+^ T cells, B cells, and plasma cells (Johncilla et al., 2015).

Although liver biopsy is not necessary for routine diagnosis of IMH, some studies indicate that it may be helpful in patients with atypical presentation or unusual clinical course, as well as a differential diagnosis in patients with viral hepatitis or autoimmune hepatitis (Haanen et al., 2017). It has been reported that liver biopsy was able to differentiate the hepatitis C virus (HCV) or IMH in HCC patients with untreated HCV, as HCV appears to have lymphocytic infiltration. In contrast, IMH appears to involve a mixed inflammatory infiltrate comprising eosinophils, histiocytes, and lymphocytes (Hsu et al., 2020). Furthermore, IMH shares several histopathological similarities with idiopathic autoimmune hepatitis (iAIH), such as panlobular inflammation, necrosis, and lymphocytic infiltrate. However, there are also exist some significant differences between IMH and iAIH, which has been reported that there is an increased presence of CD8^+^ T cells and fewer CD20^+^ B cells and CD4^+^ T cells in IMH compared to iAIH, and the panlobular inflammation is often confined in zone 3 in IMH (Kim et al., 2013; Zen and Yeh, 2018). Those findings may help differentiate IMH from iAIH.

The concern is that IMH caused by different ICIs has distinct histopathological patterns. Anti-CTLA-4 drugs are mainly characterized by specific patterns of granulomatous hepatitis, fibrin deposits, and central vein endothelialitis. However, histological findings in anti-PD-1/PD-L1 drugs are more heterogenous, of which biopsy mainly shows lobular hepatitis, periportal activity, and centrilobular necrosis (De Martin et al., 2018). Further study of the histopathological characteristics of different ICIs may be helpful in elucidating the underlying mechanisms of IMH and finally benefit the clinical practice.

### Grading criterion

The criterion of IMH grading is crucial as the severity of IMH corresponds to the management. Currently, two grading criterions, CTCAE and Drug-induced liver injury network (DILIN), are clinically used to evaluate IMH (Table 4). Both grading systems consider the alteration of serum liver enzymes and bilirubin, while most oncology clinical trials prefer to use the CTCAE grading system to evaluate the irAEs caused by ICIs, which classifies the severity as 5 grades and grade 5 refers to fatal IMH. However, it should be noted that this grading system may sometimes be insufficient to reflect the clinical severity of IMH (Personeni et al., 2021). The CTCAE system may overestimate the severity of IMH compared to DILIN. For example, transaminases >20× ULN without coagulation derangement are considered a grade 4 adverse event, which corresponds to a life-threatening event, while a normal coagulation function may not be considered a severe liver injury clinically. Therefore, compared to CTCAE, the DILIN system seems more comprehensive as it considers the international normalized ratio, symptoms, and other organ failures (Fontana et al., 2009). However, neither criteria are formulated explicitly for IMH grading but rather for elevated liver function induced by any treatment. Furthermore, which criterion is more suitable for predicting the prognosis of IMH is also unknown and still needs further exploration.

**TABLE 4:** Grading assessment of immune-mediated hepatitis by common terminology criteria of adverse events and drug-induced liver injury network.

Grade	Common terminology criteria of adverse events version 5.0 (NCI, 2017)	Drug-induced liver injury network (Fontana et al., 2009)
Grade 1	ALT>3.0 × ULN if baseline is normal; >1.5–3.0 × baseline if baseline was abnormal	Elevated serum ALT and/or ALP; TBil <2.5 mg/dl; INR <1.5; With or without symptoms (fatigue, weakness, nausea, anorexia, right upper abdominal pain, jaundice, pruritus, rash, or weight loss)
	AST>3.0 × ULN if baseline is normal; >1.5–3.0 × baseline if baseline was abnormal	
	ALP>2.5 × ULN if baseline is normal; >2.0–2.5 × baseline if baseline was abnormal	
	TBil>1.5 × ULN if baseline is normal; >1.0–1.5 × baseline if baseline was abnormal	
Grade 2	ALT>3.0–5.0 × ULN if baseline is normal; >3.0–5.0 × baseline if baseline was abnormal	Elevated serum ALT and/or ALP; TBil ≥2.5 mg/dl or INR ≥1.5 without Elevated TBil; Symptoms may be aggravated
	AST>3.0–5.0 × ULN if baseline is normal; >3.0–5.0 × baseline if baseline was abnormal	
	ALP>2.5–5.0 × ULN if baseline is normal; >2.5–5.0 × baseline if baseline was abnormal	
	TBil>1.5–3.0 × ULN if baseline is normal; >1.5–3.0 × baseline if baseline was abnormal	
Grade 3	ALT>5.0–20.0 × ULN if baseline is normal; >5.0–20.0 × baseline if baseline was abnormal	Elevated serum ALT and/or ALP; TBil ≥ 5 mg/dl with or without INR ≥1.5; Symptoms are further aggravated; Indication for hospitalization or prolonged hospitalization
	AST>5.0–20.0 × ULN if baseline is normal; >5.0–20.0 × baseline if baseline was abnormal	
	ALP>5.0–20.0 × ULN if baseline is normal; >5.0–20.0 × baseline if baseline was abnormal	
	TBil>3.0–10.0 × ULN if baseline is normal; >3.0–10.0 × baseline if baseline was abnormal	
Grade 4	ALT>20.0 × ULN if baseline is normal; >20.0 × baseline if baseline was abnormal	Elevated serum ALT and/or ALP; TBil ≥10 mg/dl or daily elevation ≥1.0 mg/dl; INR ≥1.5 with ascites, encephalopathy, or other organ dysfunction
	AST>20.0 × ULN if baseline is normal; >20.0 × baseline if baseline was abnormal	
	ALP>20.0 × ULN if baseline is normal; >20.0 × baseline if baseline was abnormal	
	TBil>10.0 × ULN if baseline is normal; >10.0 × baseline if baseline was abnormal	

ALT, alanine aminotransferase; AST, aspartate aminotransferase; ALP, alkaline phosphatase; TBil, total bilirubin; INR, international normalized ratio; ULN, upper limit of normal.

## Management

**FIGURE 2:**
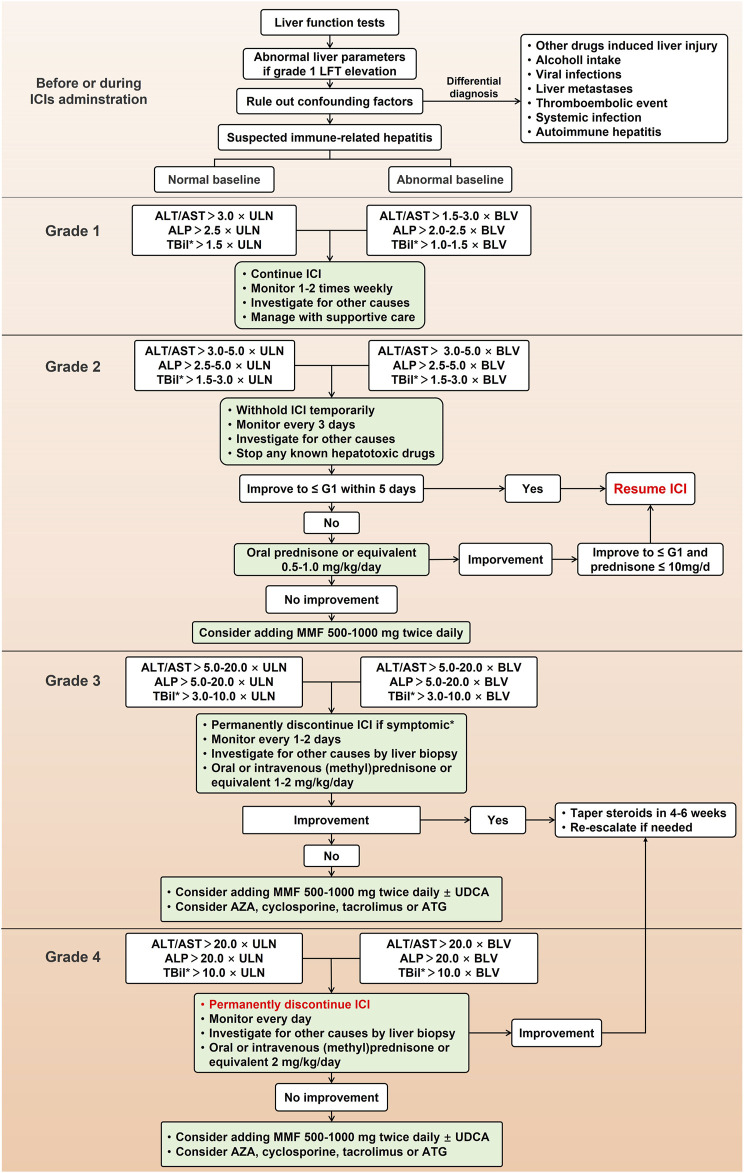
Management for immune-mediated hepatitis caused by immune checkpoint inhibitors. LFT, liver function test; ICI, immune checkpoint inhibitor; ALT, alanine aminotransferase; AST, aspartate aminotransferase; ALP, alkaline phosphatase; TBil, total bilirubin; ULN, upper limit of normal; BLV, baseline value; MMF, mycophenolate mofetil; UDCA, ursodeoxycholic acid; ATG, antithymocyte globulin; AZA, azathioprine.

Recently, American Society of Clinical Oncology (ASCO), European Society for Medical Oncology (ESMO), Society for Immunotherapy of Cancer (SITC), National Comprehensive Cancer Network (NCCN), and EASL have developed guidelines on irAEs, including IMH, to guide the management of irAEs (Haanen et al., 2017; Thompson et al., 2020; Brahmer et al., 2021; Schneider et al., 2021). Due to the lack of prospective clinical trials evaluating the effects of different treatment options, management guidelines of IMH are currently based on practice in case reports and expert consensus. Currently, most clinical practices follow the guidelines issued by ASCO in 2021, which includes the frequency of liver function tests, timing of hold and resume ICIs and corticosteroids administration. The detailed management based on current guidelines is shown in Figure 2.

Most guidelines recommend that before every ICIs administration, all patients should check liver parameters, especially for patients with a background in chronic viral hepatitis, which is recommended an antiviral therapy before the first time of ICIs therapy. To patients with asymptomatic elevations of liver tests and excluded other suspicious causes, IMH induced by ICIs should be considered. Unlike other DILI, it is not enough to discontinue the suspected culprit drugs in the management of IMH as IMH is usually induced by excessive immune response of the liver, so initiation of immunosuppressive therapy is equally necessary. Currently, all recommended management of IMH suggests using corticosteroids such as prednisone, methylprednisone, or equivalent (Miller et al., 2020). Furthermore, although CTCAE may be insufficient to reflect the clinical severity of IMH and a management algorithm based on DILIN and histopathology severity has been proposed (De Martin et al., 2018), management of IMH in most consensus and clinical trials varies with the severity of hepatitis based on CTCAE grading system. Although current guidelines of irAEs have minor differences in the management of IMH, they all follow a gradual treatment process, including continuing or ceasing ICIs, escalated first-line corticosteroids, further use of second-line mycophenolate mofetil (MMF), and the application of third-line immunosuppressive treatment (Haanen et al., 2017; Thompson et al., 2020; Brahmer et al., 2021; Schneider et al., 2021).

### Corticosteroids

Although guidelines recommend using a dosage of prednisone from 0.5 to 1 mg/kg/day for grade 2 IMH and initiating methylprednisone 1–2 mg/kg/day in more severe IMH, the timing for corticosteroid administration is still controversial. It has been shown that nearly half of patients with grade 3 or 4 IMH who discontinue ICIs can improve spontaneously without a corticosteroid treatment (Gauci et al., 2018). Another case series also reported that six patients with grade 2 or higher IMH who received no corticosteroid treatment or no escalated dose of steroid showed a sooner resolution of liver injury compared to four patients who received corticosteroids (median time: 4.7 weeks vs. 8.6 weeks) (Gauci et al., 2018), which provide a possible to avoid corticosteroids as an increased risk of severe infections are found in patients received corticosteroids during ICIs therapy (Del Castillo et al., 2016). A recent study demonstrated similar outcomes and reduced risk of corticosteroids-mediated complications of grade 3 or 4 IMH patients who received 1 mg/kg/day methylprednisolone compared to those who received high-dose steroid, which further provides support for the use of lower doses of steroids without compromising the improvement of liver function and a reduced risk of steroid-related complications (Li et al., 2022; Pan and Razumilava, 2022). In addition, budesonide, another corticosteroid used in autoimmune hepatitis, has also been reported to be effective in the treatment of grade 3 IMH and restarting ICI, which has been considered for the treatment of IMH as its metabolism feature and the lower side effects (Ziemer et al., 2017). However, the timing and indication of corticosteroid use need to be clarified further. At present, it is essential to consider an individualized treatment for IMH, and further studies are needed to evaluate the new management strategy for IMH.

### Refractory IMH to steroid

Currently, most society guidelines recommend corticosteroids as a first-line treatment for IMH. However, some cases of refractoriness on steroids were reported to not respond to steroids or failure to normalize liver function. It was recommended that if there is no response with intravenous methylprednisolone, second-line treatment of 500–1000 mg of MMF twice daily can be considered. In addition to MMF, ASCO also proposed that azathioprine (AZA) can be used as the second-line agent for steroid-refractory IMH after ruling out the infectious causes (Schneider et al., 2021), and test for thiopurine methyltransferase deficiency is required to avoid life-threatening bone marrow suppression (Ziogas et al., 2020). Although some cases reported the successful use of AZA in patients (Iwamoto et al., 2017; Huffman et al., 2018), it should be noted that the immunosuppressive effect of AZA was exerted later than that of MMF. In addition, AZA metabolites may also cause hepatotoxicity. Therefore, using AZA as a second-line treatment for IMH should be cautious.

Although MMF has been successfully used in many patients with refractory IMH to steroids, some cases still show no response after steroid and MMF treatment (McGuire et al., 2018; Motomura et al., 2020; McIlwaine et al., 2022). Therefore, given the mechanisms underlying IMH, ESMO and EASL have proposed third-line immunosuppressive agents targeting T cells, including the calcineurin inhibitors tacrolimus and cyclosporine, as well as anti-thymoglobulin (Haanen et al., 2017; European Association for the Study of the Liver, 2019). The successful use of these agents has been reported in several cases (Huffman et al., 2018; Motomura et al., 2020; McIlwaine et al., 2022). Some other treatments have also been reported to be used in both steroids and MMF refractory IMH, such as tocilizumab (Stroud et al., 2019) and plasma exchange (Riveiro-Barciela et al., 2019). Furthermore, one study suggested that treatment with ursodeoxycholic acid (UDCA) and bezafibrate should be considered in steroid-refractory IMH cholestatic injury, which may reduce the immune response *via* the proliferator-activated receptor-α-nuclear factor-κB signal pathway (Onishi et al., 2020). Some case reports showed that anti-TNF inhibitor infliximab improved hepatitis in patients with steroid-refractory IMH (Cheung et al., 2019; Corrigan et al., 2019). However, considering the potential hepatotoxicity, all guidelines do not recommend its use in IMH. A detailed additional treatment for steroid-refractory IMH in case reports showed in Table 5.

**TABLE 5:** Additional treatments for steroids-refractory immune-mediated hepatitis from case reports.

Additional treatments	Time for recovery of liver parameters	Ref
MMF 1 g/day for 1 week plus ATG 1.5 mg/kg/day for 2 consecutive days	1 month from the start of the ATG	Chmiel et al. (2011)
MMF 500 mg twice-daily plus intravenous ATG 1.5 mg/kg/day for 2 consecutive days	After 49 days	Motomura et al. (2020)
MMF 1 g/day plus two intravenous doses of ATG of 100 and 50 mg for 2 consecutive days	After 162 days	McGuire et al. (2018)
MMF 1 g twice daily for 2 weeks plus ATG for 2 dosages	After 2 eeks	Ahmed et al. (2015)
ATG 100 mg/day	After 5 days	Spankuch et al. (2017)
MMF 500 mg plus tacrolimus 500 mg twice daily	After 186 days	McIlwaine et al. (2022)
MMF 1 g twice daily plus tacrolimus 5 mg/kg/day	N/A	Cheung et al. (2019)
MMF 1 g plus tacrolimus 1.5 mg/kg twice daily	After 9 weeks	Ziogas et al. (2020)
Cyclosporine 100 mg twice daily	After 40 days	Huffman et al. (2018)
Oral AZA with 100 mg/day	After 1 month	Iwamoto et al. (2017)
UDCA 600 mg/day plus bezafibrate 400 mg/day	After 35 days	Onishi et al. (2020)
Oral MMF 2 g/day plus UDCA	After 56 days	Doherty et al. (2017)
MMF 1.5 g/day plus plasma exchange (1,500 ml of 5% albumin plus 4 units of plasma, every other day)	After 2 weeks	Riveiro-Barciela et al. (2019)

MMF, mycophenolate mofetil; ATG, anti-thymocyte globulin; AZA, azathioprine; UDCA, ursodeoxycholic acid.

### Withhold and resume ICIs

Another controversial point in the treatment of IMH is whether to permanently cease or resume ICIs in patients with grade 3 or 4 IMH. As current guidelines recommend, ICIs should be permanently ceased in patients with more severe IMH (grade 3 or 4). However, according to a systemic review, grade 3 or 4 IMH should be considered to resume ICIs therapy or switch CTLA-4 inhibitors to PD-1/L1 inhibitors when hepatitis severity improves to grade 2 (Peeraphatdit et al., 2020). Furthermore, another study reported that four patients with grade 3 or 4 IMH were successfully given further immunotherapy after improved liver function, which provides the possibility of resuming ICIs in patients with more severe IMH (Cheung et al., 2019). However, a prospective multicenter study reported that retreatment with ICIs in patients with previous grade 3 or 4 IMH led to 8 of 23 recurrences (Riveiro-Barciela et al., 2022). Moreover, the administration of budesonide during resuming ICIs was considered another promising treatment in patients with severe IMH (Ziemer et al., 2017). In summary, some arguments still exist in the management of IMH. With the understanding of IMH evolved over the years, individualized management should be considered, and the underlying mechanisms of IMH should be further explored to set out a more appropriate management guideline.

## Prognosis

Most patients with IMH can recover spontaneously or after corticosteroid administration. For the recovery time, it has been reported that IMH usually resolves in 5–9 weeks (Gauci et al., 2018). However, extended time of ALT levels returned to normal have also been reported in several cases, especially in steroids refractory IMH (Matsubara et al., 2018; McGuire et al., 2018; McIlwaine et al., 2022), which may due to a more severe IMH in these cases. Considering this, a timely diagnosis and management of IMH are critical for prognosis. Nonetheless, there are few studies to validate the recovery time of IMH with different severity and treatments, which may be a direction for selecting treatment and prognosis prediction of patients with IMH in the future. Furthermore, for the mortality of IMH, a retrospective multicenter review showed that the incidence of fatal IMH was 0.01% (5/3345) of all patients treated with ICIs. However, IMH accounted for a high proportion of fatal cases (23.8%, 5/21) (Wang et al., 2018). Moreover, other studies also reported a high mortality rate for IMH (Vozy et al., 2019). These results suggest that attention should be paid to IMH, especially fatal cases, and with the development of diagnosis and management of IMH, the mortality of IMH should be reevaluated.

As to the oncology outcomes, fewer studies have focused on the clinical outcomes of IMH compared with other irAEs. Despite this, a study showed an excellent outcome and overall survival in patients with IMH (Patrinely et al., 2021), which is consistent with the results from studies in extra-hepatic irAEs (Abu-Sbeih et al., 2018; Das and Johnson, 2019; Quach et al., 2019). Although another study indicated that patients with previous IMH showed a lower tumor response and poorer survival outcomes, these results may be due to liver metastases and the administration of other treatments for advanced cancer rather than ICIs (Tsung et al., 2019). A retrospective study reported that IMH was not associated with anti-tumor efficacy and overall survival in patients treated with ICIs (Yamamoto et al., 2021). Moreover, studies have shown no difference in survival outcomes between IMH patients with and without steroid treatment (Gauci et al., 2021). Therefore, given that the oncology outcome of IMH is controversial, more extensive prospective studies are needed to evaluate the prognostic impact of IMH.

## Future prospectives

With the success of ICIs in several types of cancer, more and more patients are being treated with ICIs. However, ICIs therapy also causes a variety of irAEs. Due to the immunological characteristics of the liver, ICIs also cause liver-related adverse events, usually termed “immune-mediated hepatitis”. Although IMH is not common compared with other irAEs, with the expanded indications of ICIs therapy, an increasing number of cases diagnosed with IMH are reported. IMH has become increasingly concerned about its potential influence on anti-tumor therapy and lethality. However, the diagnosis and management for IMH are based on found in retrospective case series experience, so there is an urgent need for some randomized clinical trials to clarify the current debate in the IMH, such as further exploring the molecular mechanisms and identifying the prediction markers of IMH as well as evaluate the role of liver biopsy in IMH causality and grading assessment. In addition, we need prospective studies investigating steroid and non-steroid based management of IMH to determine the ideal treatment regimen and better delineate the threshold for appropriate treatment rechallenge after initial management.
